# An Overview of Vascular Compression Syndromes and Associations with Autonomic Dysfunction: A Review

**DOI:** 10.3390/biomedicines14030689

**Published:** 2026-03-17

**Authors:** Brandon M. Davis, Petra Rantanen, Grace Seo, Siya Thadani, Elizabeth B. Spencer, Edward Hepworth, Alexis Cutchins

**Affiliations:** 1 Emory Clinical Cardiovascular Research Institute, Division of Cardiology, Emory University School of Medicine, Atlanta, GA 30322, USA; 2 Department of Medicine, Emory University School of Medicine, Atlanta, GA 30322, USA; 3 Emory University, Atlanta, GA 30322, USA; 4 Minimally Invasive Procedure Specialists, Highlands Ranch, CO 80130, USA; 5 Western Sinus and Skull Base Consultants, LLC, Denver, CO 80206, USA; 6 Cutchins Cardiovascular Medicine, New York, NY 10022, USA

**Keywords:** dysautonomia, vascular compression syndrome, postural orthostatic tachycardia syndrome, hypermobility spectrum disorders

## Abstract

**Background**: Vascular compression syndromes are increasingly recognized as underdiagnosed contributors to morbidity in patients exhibiting dysautonomia. Underlying vascular compression syndromes affecting the head and neck, abdomen, pelvis, and lower extremities may influence venous return, neurohormonal signaling, and autonomic regulation. There is considerable clinical overlap among these syndromes, as well as between hypermobility spectrum disorders (HSD) and dysautonomia, indicating possible shared or interacting pathophysiological mechanisms. **Purpose/Aims**: This hypothesis-generating narrative review synthesizes current evidence linking vascular compression syndromes with dysautonomia, highlights potential mechanistic pathways, identifies patterns of syndromic overlap, and emphasizes the importance of systematic evaluation in affected patient populations. **Key Findings**: Evidence from retrospective studies, case series, and clinical observations indicates that vascular compression syndromes may be prevalent among patients with dysautonomia, particularly postural orthostatic tachycardia syndrome (POTS) and HSD, yet are often unrecognized. Proposed mechanisms based on limited data include impaired venous capacitance and preload reserve, increased intracranial pressure, altered renin–aldosterone and cortisol signaling, underlying autoimmune and systemic diseases, and sympathetic ganglion irritation. Several compression syndromes show symptom overlap and frequent co-occurrence, especially in patients with connective tissue disorders. Emerging data suggest that targeted interventions, such as surgical decompression or venous stenting, may improve orthostatic intolerance and quality-of-life measures in selected patients, though high-quality prospective data remain limited. **Conclusions**: Vascular compression syndromes may be an important yet underappreciated contributor to dysautonomia. Increased clinical awareness and systematic screening may reduce diagnostic delays and morbidity in this underserved population. Prospective studies are needed to clarify prevalence, establish causal relationships, and determine the impact of targeted treatments on autonomic outcomes.

## 1. Introduction

Vascular compression syndromes are underdiagnosed conditions that can occur in patients with dysautonomia [1,2,3,4,5,6]. Some of these syndromes, including nutcracker syndrome (NCS) and May-Thurner syndrome (MTS)/Non-thrombotic iliac vein lesion (NIVL), have been postulated to directly cause or exacerbate symptoms of dysautonomia through theoretical mechanisms such as decreased cerebral perfusion pressure, decreased venous return to the heart, and changes in cortisol secretion and autonomic function secondary to alterations in the renin-aldosterone system [1,6,7,8]. There is significant overlap among different vascular compression syndromes, particularly in patients with hypermobility spectrum disorders (HSD) [9]. There is also a substantial intersection between HSD and dysautonomia [10]. As such, vascular compression syndromes can be considered as a mechanism or an exacerbating factor for dysautonomia in the HSD population.

This theoretical, hypothesis-generating, narrative review will describe multiple vascular compression syndromes, covering internal jugular venous stenosis (IJVS), thoracic outlet syndrome (TOS), median arcuate ligament syndrome (MALS), NCS, superior mesenteric artery syndrome (SMAS), MTS/NIVL, and chronic venous disease (CVD), with particular attention to their links to dysautonomia and HSD. We use “dysautonomia” to refer broadly to autonomic dysfunction, which encompasses phenomena such as postural orthostatic tachycardia syndrome (POTS) and orthostatic intolerance (OI). When specific disorders or symptoms have been referred to in the literature, we will use the more specific term. Similarly, we use “HSD” to refer broadly to joint hypermobility, but we will use more specific terms, such as “hypermobile Ehlers-Danlos Syndrome (hEDS),” when they are used in the literature. Ultimately, we recommend evaluating the HSD patient population for vascular compression syndromes, especially considering the significant impact that these underrecognized syndromes can have on morbidity for this underserved population.

## 2. Methods

A narrative literature review was conducted to identify existing literature and evidence regarding various vascular compression syndromes and their relationship to dysautonomia. A literature search was conducted using electronic databases, including PubMed Central, MEDLINE, and Scopus, using the terms “dysautonomia,” “autonomic dysfunction,” “orthostatic intolerance,” and the accompanying primary MeSH term, “Autonomic Nervous System Diseases,” in combination with Boolean operators (AND/OR) for each vascular compression syndrome (“internal jugular venous stenosis,” internal jugular venous compression,” thoracic outlet syndrome(s),” “nutcracker syndrome,” “median arcuate ligament syndrome,” “superior mesenteric artery syndrome,” “superior mesenteric artery compression,” “May-Thurner syndrome,” “non-thrombotic iliac vein lesion,” “chronic venous insufficiency,” and “chronic venous disease”). To identify associations with HSD, we used the previous search terms in combination with the Boolean operator (AND) and the following terms: “hypermobility spectrum disorder,” “Ehlers-Danlos syndrome,” “hypermobile Ehlers-Danlos Syndrome,” and “collagen vascular disorders.” Additional relevant articles were identified through manual review of reference lists. Studies included in this review were published between 2010 and 2026 and involved one or more vascular compression syndromes and prevalence or outcome data related to autonomic dysfunction. In rare cases, studies published before 2010 were included due to limited data. Selected studies were reviewed and synthesized to reveal key themes, emerging patterns, and gaps in the literature.

## 3. Internal Jugular Venous Stenosis

Significant symptomatic disease in IJVS occurs when extrinsic compression of the jugular veins obstructs venous flow to the heart and increases intracranial pressure (ICP) (Table 1) [11,12,13,14,15]. Given the paucity of smooth muscle and elastic fibers in the venous system, the jugular veins are relatively vulnerable to extrinsic compression from multiple structures, including bones, muscles, fascia, ligaments, lymph nodes, and other blood vessels (i.e., a tortuous internal carotid artery). Common symptoms include non-specific neurologic, head, and neck findings, such as headache, dizziness, neck discomfort, hearing loss, tinnitus, and blurry vision [11].

**Figure 1:**
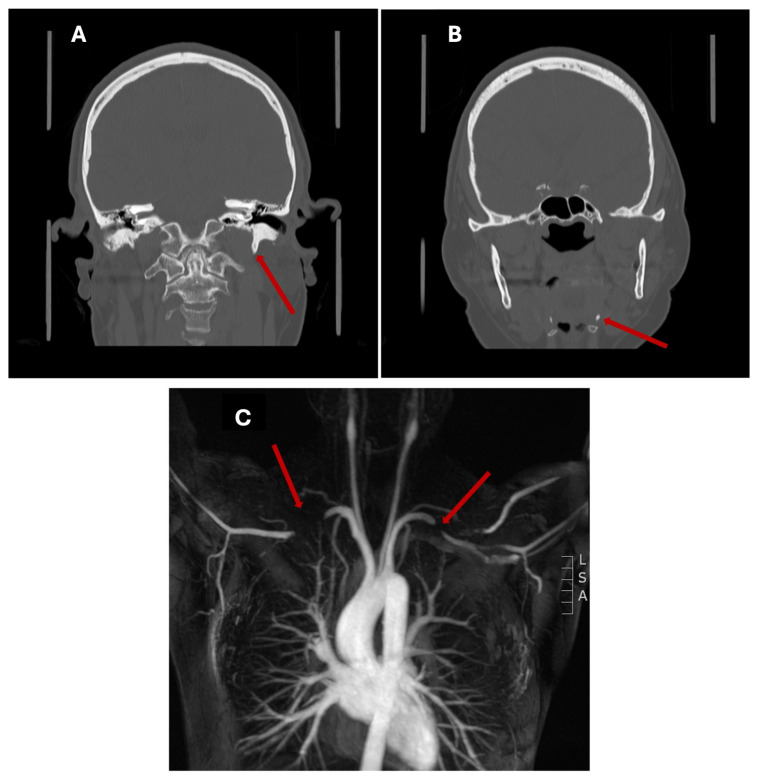
Cervical and Thoracic Compression Syndromes. (**A**,**B**) Styloidogenic compression (Eagle syndrome): an elongated styloid process originating from the mastoid process ((**A**), red arrow) and terminating near the hyoid bone ((**B**), red arrow) predisposes to compression of vascular structures (Modified from Dr. Omar Giyab, Radiopaedia.org, rID: 95989). (**C**) Arterial thoracic outlet syndrome: MRA depicting thoracic outlet syndrome resulting in stenosis and compression of the subclavian artery unilaterally or bilaterally (red arrows) (Modified from Dr. Daniel Fascia, Radiopaedia.org, rID: 47517).

IJVS may occur from several different anatomical processes. Most commonly, it results from compression by an anatomic variant, such as an elongated or ossified styloid process of the temporal bone and the C1 transverse process, often referred to as styloidogenic jugular stenosis (Eagle Syndrome) (Figure 1). Hypertrophy of surrounding muscles, such as the scalene, sternocleidomastoid, and omohyoid muscles, as well as downward displacement of the first rib, can also cause IJV compression. Similar mechanisms are observed in TOS, suggesting shared pathophysiology [30]. The IJV may also be compromised by ligamentous cervical instability, a degenerative process that occurs as chronic stretching of ligaments leads to loss of lordotic curvature, displaces C1 anteriorly, and compresses the IJV. This pathology has been proposed to be associated with vagal nerve degeneration, although there is no experimental literature that has substantiated this mechanism [31,32].

Jugular stenosis can also occur dynamically with neck rotation or head flexion at C1–3 and has been posited to affect ICP, although no definitive studies have explained this phenomenon [33,34]. Moreover, chin flexion and jaw movement may influence jugular gradients in patients with suspected venographic intracranial hypertension [33]. Local gradients developing in the neck have been shown to affect upstream venous sinus pressures. However, similar gradients and stenoses have been observed in asymptomatic patients. It is unclear why some patients with similar degrees of stenosis lack symptoms, but this could reflect differential sensitivities to venous flow abnormalities [35]. Symptoms are hypothesized to develop from insufficient internal jugular outflow with adequate extrajugular flow, adequate jugular outflow but inadequate collateralized flow, or impairment in both jugular and extrajugular flow [35]. Although not experimentally validated, these outflow abnormalities are hypothesized based on known physiologic mechanisms of cerebral venous outflow and venous flow equations.

Beyond extrinsic compression, infectious and inflammatory conditions are known to cause IJVS due to intraluminal thrombosis and fibrotic remodeling. Post-anginal infection leading to IJV thrombophlebitis, also known as Lemierre’s syndrome, may occur through lymphangitic or hematogenous spread, contiguous spread through the loose connective tissue of the pharynx, and through pathogen-mediated mucosal alterations. *Fusobacterium necrophorum*, *Salmonella paratyphi*, and *Actinomyces*, among other bacterial species, have been implicated in the disease [36,37]. Epstein-Barr virus (EBV) has also been linked to Lemierre’s syndrome through case reports, but a larger study found a low incidence of EBV in patients with the syndrome [38]. Given the discrepancy in disease incidence between smaller- and larger-scale studies and the limited available literature, upper respiratory viruses may not play a significant role in disease development, but their pro-inflammatory effects warrant further study. Chronic cervicofacial actinomycosis has been shown to cause IJVS through inflammatory mass effect and post-thrombotic fibrotic remodeling [39]. Lastly, various rheumatologic conditions have been linked to JVS through mechanisms such as vessel wall inflammation in Behçet’s disease and Sjogren syndrome, thrombosis in antiphospholipid antibody syndrome, and IJV encasement by fibrotic masses in immunoglobulin G4-related disease [40,41].

Treatment of IJVS is dependent on the etiology of the obstruction. Options include styloidectomy, resection of the lateral mass of the C1 vertebra, IJV stenting, angioplasty, or mastoidectomy [11]. Other interventions aim to reduce increased ICP, a common complication of IJVS, through non-surgical management with diuretics such as acetazolamide or surgical management with CSF shunting or transverse venous sinus stenting [11]. Additional nonoperative management may include anticoagulation for thrombosis, chemical denervation injections [42], and prolotherapy for ligamentous cervical instability [32], whereas autologous stem cell therapy is a potential future therapy for ligamentous regeneration. Overall, data on the optimal treatment for IJVS are currently limited to case reports and case series, and further studies are needed to determine the most effective treatments [1].

At present, there are no clear data supporting a direct relationship between jugular venous stenosis and dysautonomia, despite abundant anecdotal evidence linking the two conditions. IJVS has been hypothesized to be associated with disruptions in cerebral venous outflow, leading to increased ICP and dysregulated venous return, a pattern also observed in POTS [1]. Specifically, IJV compression may result in nodose ganglion crowding and hypersensitivity at the jugular foramen, affecting baroreceptor responsiveness [43]. As previously described, some structural abnormalities have been hypothesized to induce vagal nerve degeneration and subsequent autonomic symptoms, but there is little corroborating evidence [32]. Other underlying conditions, such as collagen vascular disorders, have been associated with this condition, but the evidence based is quite limited. One case series evaluating jugular venous stenting for symptomatic disease found that 69% had a pre-existing connective tissue disorder [44]. However, further studies are needed to elucidate pathophysiological mechanisms.

## 4. Thoracic Outlet Syndrome

TOS is a group of disorders caused by compression of the thoracic outlet and its accompanying neurovascular structures, leading to arm pain, swelling, fatigue, paresthesia, weakness, and hand discoloration [45]. The thoracic outlet comprises the interscalene triangle, costoclavicular space, and pectoralis minor space, where compression may occur in any one or more of these spaces as part of the cervicoaxillary tunnel [46,47]. While this syndrome may be present at rest, it is typically associated with more profound neurological and/or vascular deficits related to arm abduction and overhead movements. TOS is categorized into neurogenic TOS (nTOS) and vascular TOS, comprising venous (vTOS) and arterial (aTOS) etiologies [48]. nTOS is the most common form (95% of cases), characterized by constriction of the brachial plexus cords [45,49], resulting in significant neurological symptoms on the affected side without a distinct peripheral neurological distribution. Vascular TOS is much rarer (3–5% of cases) and is characterized by constriction of the axillary or subclavian artery or vein, which produces predominantly vascular symptoms such as pain, swelling, and skin color changes (Figure 1).

TOS is diagnosed largely through history and physical exam, while imaging studies confirm the diagnosis and identify compression sites. Physical exam provoking maneuvers, such as over-the-head movements, are suggestive but should be interpreted in the context of underlying symptoms and history. Initial diagnostic imaging should include a plain radiograph to evaluate for cervical ribs, followed by duplex ultrasonography to assess arterial or venous flow abnormalities with provocative maneuvers. Traditionally, the best non-invasive test for nTOS and vTOS is MRI with vessel imaging (Table 1) [16,17,50]. Ultimately, venography and IVUS, with and without provocative measures, will likely become the gold standard for diagnosis and are increasingly being used in clinical practice. Non-invasive treatments for TOS include anti-inflammatory medication, weight loss, physical therapy, and botulinum toxin injections [45]. Botulinum toxin injections are associated with a positive response [48]. Surgical treatments include brachial plexus decompression, neurolysis, and scalenectomy with or without first rib removal [45]. Stenting of the subclavian vein is generally not considered appropriate due to the high rate of stent fracture and failure in this location. However, residual stenoses following surgical decompression may respond to angioplasty, while angioplasty pre-decompression has shown no benefit [51].

While nTOS is the most widely studied form of TOS, aTOS and vTOS are largely underrecognized conditions that may be more common in patients with POTS or HSD and warrant further dedicated study. HSD is known to affect connective tissue, leading to increased fragility and anatomical features that favor entrapment syndromes, providing a theoretical basis for an association between HSD and TOS, but there is a lack of data indicating a clear association [52,53]. Regarding dysautonomia, in a retrospective study of 64 patients undergoing evaluation for TOS or brachial plexus dysfunction at a chronic fatigue clinic, all but one had OI, and 46 met criteria for POTS, implying a significant association [2]. In contrast, in a study of 1142 patients with POTS, 40 had symptoms of TOS, and 20 (1.75%) tested positive for vascular TOS. nTOS was not evaluated [13]. The heterogeneity in the association between dysautonomia and TOS seen in the smaller subspecialty cohort and the larger cohort is a limitation of the current evidence. It underscores the need for more prospective studies with larger sample sizes investigating the association between dysautonomia and TOS. It has also been hypothesized that biomechanical strain in the brachial plexus may influence heart rate via neural connections with the stellate ganglion, the intrathoracic nerves, and the second and third thoracic sympathetic ganglia [2], but further research is needed to assess this connection.

## 5. Median Arcuate Ligament Syndrome

**Figure 2:**
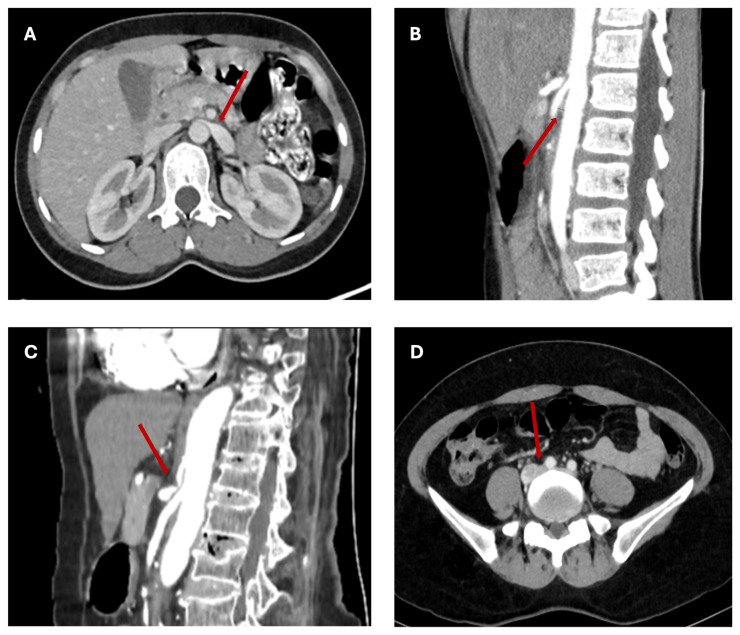
Abdominal and Pelvic Compression Syndromes. (**A**) Nutcracker syndrome: the left renal vein is stenotic due to a reduced aortic-mesenteric angle (mesenteric artery superior, aorta posterior), leading to pathological compression (red arrow) (Modified from Dr. Abdulkhaleq A. Bin Nuhaid, Radiopaedia.org, rID: 196188). (**B**) Superior mesenteric artery syndrome: significantly reduced aortic-mesenteric angle (~15 degrees) (red arrow, dotted line) that results in upstream duodenal compression (Modified from Dr. Mohammad Farghali Ali Tosson, Radiopaedia.org, rID: 55941). (**C**) Median arcuate ligament syndrome: “hook-shaped” (red arrow) appearance of the celiac trunk and post-stenotic dilation due to an enlarged median arcuate ligament (Modified from Dr. Rasha Karam Mahmoud Mohammed, Radiopaedia.org, rID: 178113). (**D**) May-Thurner syndrome: focal stenosis and post-stenotic dilation of the left common iliac vein due to compression by the right common iliac artery (superior) and 5th lumbar vertebral body (posterior) (Modified from Dr. Thành Anh Lê, Radiopaedia.org, rID: 210405).

MALS, also known as Dunbar syndrome, refers to compression of the celiac trunk and plexus by the median arcuate ligament (MAL) (Figure 2). Symptoms include nausea, vomiting, diarrhea, unintentional weight loss, postprandial abdominal pain, and extreme aversion to food [54]. Vascular insufficiency due to celiac trunk compression is traditionally thought to cause most symptoms [54], but newer literature suggests that MALS is largely a neurogenic disease with neuropathic-mediated pain [54,55,56].

MALS may be diagnosed through non-invasive and invasive methods (Table 1) [17,19,20,21,22]. Abdominal Doppler ultrasound can detect functional flow changes of the celiac trunk [57]. Additionally, lateral aortic angiography can demonstrate asymmetric focal narrowing of the proximal celiac axis and its variation during the respiratory cycle [58]. Celiac plexus block is an additional diagnostic method for MALS, and symptomatic improvement following neuronal blockade is suggestive of a primary neuropathic etiology of pain [59].

The main treatment for MALS is surgical release of the MAL, including robotic, laparoscopic, and open techniques, as well as celiac ganglionectomy and celiac artery revascularization [54,60,61]. As above, celiac plexus and splanchnic nerve blocks are both diagnostic and therapeutic strategies. Effective celiac plexus nerve blocks may also predict a treatment response to MAL surgical release, as a single-center retrospective analysis of a subset of patients who underwent successful celiac plexus block showed positive clinical outcomes following surgery (96% of patients) [62].

Several small case series have reported an overlap in diagnoses of MALS and POTS, ranging from 27–50% [54,63]. These studies, among others, suggest an association between celiac plexus irritation in MALS and dysautonomia symptoms. In addition, there is a small body of literature investigating procedural outcomes in patients with both MALS and POTS or OI. A prospective study of 31 pediatric patients with OI/POTS and MALS undergoing MAL release demonstrated improvement in POTS symptoms in about half of the participants. Average follow-up was 22 months [64]. A smaller study of MAL release in twelve patients with MALS did not specifically assess the impact of treatment on co-occurring dysautonomia, but 50% of included patients had POTS [65]. A splanchnic nerve block was shown to be effective in a case series of pediatric patients with POTS and chronic abdominal pain [64]. Moreover, in a small, retrospective single-center study of 77 celiac plexus and retrocrural splanchnic nerve blocks for non-cancer-related pain, 77% of patients with POTS/dysautonomia and 70% of patients with MALS experienced symptomatic relief for a mean duration of approximately 40 days [66]. While these procedural studies do not establish a directional relationship between MALS and dysautonomia or include a proposed mechanism, the significant overlap between MALS and dysautonomia in participants, and in some studies, indicates a potential relationship that warrants further study.

## 6. Nutcracker Syndrome

Nutcracker syndrome (NCS) is the symptomatic compression of the left renal vein (LRV) between the superior mesenteric artery (SMA) and the aorta (Figure 2). In contrast, nutcracker phenomenon (NCP) refers to imaging findings of LRV compression without symptoms. In NCS, LRV compression results in renovascular hypertension, which can subsequently lead to the characteristic symptoms of flank pain and hematuria. Additionally, an incompetent left gonadal (or ovarian) vein can decompress the system, resulting in varices that can cause pelvic pain and ovarian vein varices in women, or varicocele in men [67,68]. However, the presentation can be variable with proteinuria, abnormal uterine bleeding, nausea, and anemia as additional symptoms [68]. NCS has also been linked with new daily persistent headache. This has been hypothesized to result from LRV compression inducing collateral flow from the ascending lumbar vein into the transverse lumbar veins and the epidural plexus [69]. This may also lead to elevation of cerebrospinal fluid pressure, resulting in headache [70,71].

NCS is diagnosed by a combination of clinical symptoms and imaging findings [67,68]. There is no gold standard for an imaging modality in the diagnosis of NCS, but Doppler ultrasound, CT, MRI, and contrast venography all play a role [67]. Parameters include degree of LRV stenosis, aortomesenteric angle, and peak systolic LRV velocity, where suggested cutoffs have been identified (Table 1) [21,22,23,24,25]. It is also important to note that LRV compression often increases when the patient is upright, so imaging studies performed in the supine position may underestimate LRV stenosis [68]. Treatment options include conservative management emphasizing weight gain as well as open or endovascular interventions [67]. NCS and NCP are considered rare, with an unknown exact prevalence. However, a recent single-center study demonstrated a prevalence of 30% and 15% of NCP and NCS, respectively, in a sample of 1223 renal patients who underwent Doppler US examinations. NCP was defined as peak flow velocity > 100 cm/s at the aortomesenteric LRV, suggesting that the prevalence may be higher than previously thought [72].

In addition to the symptoms described above, symptoms of dysautonomia have been linked to NCS, largely in the pediatric population. In a sample of 53 pediatric patients with NCP (diagnosed by a 5:1 distended-to-stenosed LRV ratio on US plus CT and/or MRA), 42% were diagnosed with OI and reported symptoms such as dizziness, syncope, palpitations, malaise, and headaches [8]. In another sample of 45 pediatric patients with NCP (diagnosed by US with an aortomesenteric/renal hilum peak velocity ratio greater than 5:1, a renal hilum/aortomesenteric ratio greater than 5:1, and SMA angle smaller than 35°), 55% endorsed symptoms of orthostatic disturbances, including dizziness, fatigue, and palpitations. Twenty percent were diagnosed with POTS [3]. A case series described four adolescents with NCS (diagnosed by hematuria and US plus MRA or venography) who also exhibited orthostatic symptoms, including headache, tachycardia, and/or syncope. Two of the four had tachycardia at rest. Notably, collagen disorders were excluded from this group [7]. The authors hypothesized that these symptoms resulted from decreased venous return to the heart or from changes in cortisol secretion and autonomic function secondary to alterations in the renin-aldosterone system induced by renovascular hypertension [7,8]. However, this mechanism is purely theoretical. We found no studies that investigated improvement in OI after NCS treatment. This is an area in desperate need of study.

## 7. Superior Mesenteric Artery Syndrome

Along a similar pathophysiological pathway to NCS, SMAS refers to duodenal compression between the SMA and the aorta (Figure 2), resulting in duodenal obstruction. This is frequently caused by weight loss, resulting in loss of mesenteric fat between the SMA and the aorta. SMAS is rare, with a prevalence of 0.013–0.78% in the general population [73]. Musculoskeletal abnormalities, including spinal conditions such as scoliosis, scoliosis-corrective surgery, body casting, and significant lumbar osteophytes, are known causes of SMAS [74,75,76]. Congenital abnormalities, such as defects in the ligament of Treitz, may also cause SMAS [76]. Common symptoms include nausea, vomiting, epigastric pain, early satiety, bloating, and weight loss. Given these nonspecific symptoms, SMAS is often misdiagnosed as functional dyspepsia or anorexia nervosa. SMAS is diagnosed by characteristic symptoms and supportive radiological findings (Table 1) [21,22,23]. While traditional diagnostic criteria are based on upper GI series, SMAS is often diagnosed with CT, MRI, abdominal ultrasound, or endoscopy. Treatment is usually conservative and supportive, with emphasis on weight gain to increase mesenteric fat and decrease the degree of obstruction [76]. Surgical therapy can be done if conservative treatment fails.

Unlike other abdominal compression syndromes, no data specifically exist that support an association between SMAS and dysautonomia. Patients with POTS are known to experience GI-related symptoms due to motility issues, and some patients experience rapid weight loss due to poor nutrition [4]. This, in theory, could result from SMAS in some patients, but this has not been shown in the current literature. The lack of association between SMAS and dysautonomia may be explained by the pathophysiology being intestinal compression rather than a compression of a blood vessel, thereby lacking the theoretical mechanisms proposed in other vascular compression syndromes, such as decreased venous return or disrupted neurohormonal or autonomic signaling. Importantly, numerous case reports describe overlap between SMAS and NCS, possibly due to the shared anatomic location of the duodenum and LRV between the SMA and the aorta [77,78,79].

## 8. May-Thurner Syndrome/Non-Thrombotic Iliac Vein Lesion

The preferred term for pelvic venous compression is NIVL, although most patients are more familiar with the term “May-Thurner.” MTS/NIVL results from extrinsic compression of the left common iliac vein by an overlying right common iliac artery anteriorly and a lumbar vertebral body posteriorly (Figure 2) [80,81]. Other compressions can occur in the iliac venous system, including compression between the internal and external iliac vein confluence and the pelvic inlet due to bony compression posteriorly. Chronic pulsatile arterial compression may lead to endothelial injury, venous wall remodeling, and intimal hyperplasia. Disordered deposition of elastic fibers and collagen contributes to luminal irregularities, thereby reducing venous capacitance and impairing blood flow. The proximity of structures and consequential compression predispose affected individuals to venous outflow obstruction and an increased risk of DVT, classically referred to as MTS [82]. Current estimates suggest that approximately 2–5% of all DVTs are attributable to MTS [83,84]. However, retrospective studies have demonstrated a disproportionately higher prevalence of left-sided lower-extremity DVTs and venous insufficiency in the general population, suggesting that MTS may be substantially underdiagnosed [85].

MTS/NIVL is associated with specific epidemiological risks and clinical features. The condition occurs almost exclusively in female patients, a finding hypothesized to be related to accentuated lumbar lordosis and a narrower pelvic space [86]. The condition often manifests following surgery or during the peripartum period from compression from a gravid uterus, consistent with an inciting phenomenon in which venous compression is compounded by a transient hypercoagulable state [84,87]. Traditionally, and likely erroneously, iliac compression has been associated with unilateral lower extremity swelling of the left leg, accompanied by skin changes such as hyperpigmentation or hypopigmentation, warmth, erythema, and pain [82]. However, recent data challenge this conception. An analysis of 271 patients with OI and pelvic venous disease who were treated with iliac vein stenting for NIVL showed that bilateral lower extremity pain was present in 55% percent of patients and swelling in only 40% of patients. Isolated, unilateral left-leg symptoms were present in only 13% of patients, and 17% had no lower extremity symptoms [29]. These data suggest that lower extremity findings in MTS/NIVL are grossly overstated and poorly understood. If left untreated, MTS/NIVL may lead to chronic venous insufficiency, skin discoloration, venous ulceration, persistent pain, post-thrombotic syndrome, and, in rare cases, chronic thromboembolic pulmonary hypertension [81].

MTS/NIVL is diagnosed with venous imaging (Table 1) [23,24,26,27,28,29]. Venous duplex ultrasonography is recommended as the first-line imaging modality due to its wide availability and lack of ionizing radiation. However, ultrasonography has limited sensitivity for evaluating pelvic veins proximal to the inguinal ligament, a region implicated in many cases of MTS/NIVL and accounting for approximately 20% of DVT cases [88]. In such cases, attenuated venous flow velocities or nondiagnostic findings should prompt further evaluation with advanced imaging [89]. Magnetic resonance venography (MRV) offers several advantages for evaluating MTS/NIVL and other abdominal vascular compression syndromes, including high spatial resolution, detection of subtle luminal abnormalities, and comprehensive assessment of surrounding anatomic structures [24]. MRV demonstrates pooled sensitivity and specificity of 92% and 95%, respectively, for DVT detection [88]. Comparative studies have shown that MRV has accuracy comparable to conventional venography in assessing the severity of iliac vein stenosis [90]. Computed tomography venography is an alternative modality but is generally less favored due to contrast exposure and lower soft tissue resolution.

Management of MTS/NIVL is varied. Acute thrombotic presentations are optimally treated with catheter-directed thrombolysis or thrombectomy [91,92,93]. However, most patients present with chronic symptoms rather than acute thrombosis. Iliofemoral vein stenting is a safe and effective intervention for symptomatic MTS as well as other causes of iliofemoral venous obstruction [94,95,96,97]. In a randomized controlled trial of 51 patients with symptomatic iliac vein obstruction (>50% stenosis by intravascular ultrasound), stent placement achieved 100% procedural success. It was associated with high six-month patency rates and significant improvements in pain and quality-of-life measures compared with standard medical therapy [98]. A large meta-analysis evaluating the safety and efficacy of stent placement for non-thrombotic, acute thrombotic, and chronic post-thrombotic iliofemoral venous disease reported success rates of 94–96% and relatively low complication rates (0.3–1.1% for major bleeding, 1–6.8% for early thrombosis) [97]. Anticoagulation is required for at least six months in cases of acute DVT, while the role of anticoagulation and antiplatelet therapy after angioplasty and stenting for NIVL remains variable and is typically individualized based on bleeding risk, although recent consensus guidelines recommend no treatment in patients who undergo stenting for NIVL without any other indication [86,99,100].

In addition to venous manifestations, patients with MTS/NIVL may experience symptoms consistent with autonomic dysfunction, including lightheadedness, brain fog, fatigue, weakness, tachycardia, and exercise intolerance. These symptoms could be related to orthostatic stress, which reduces cardiac filling pressures and preload reserve, and compensatory mechanisms may fail to adequately augment cardiac output [5]. The underlying disease process is unclear, but possibly multifactorial, involving partial dysautonomia and OI from neuropathic processes, as well as relative hypovolemia from venous pooling in these patients. Data on venous pooling are limited and largely derived from small studies. For example, several small experimental studies have shown enhanced sympathetic responses with larger volumes of infused saline and greater venous distention in the upper extremities than with lower volumes [101,102]. Impaired peripheral and splanchnic vasoconstriction is a known mechanism of partial dysautonomia in patients with POTS, in which regional differences in sympathetic nerve function are more pronounced in the legs, with inadequate norepinephrine signaling [4,6,103]. Moreover, some patients with MTS/NIVL may experience pelvic pain due to venous congestion, where chronic pelvic pain may present with cardio-venous symptoms, like exertional dyspnea and impaired exercise tolerance [104]. A clinical trial comparing healthy controls to patients with various bladder pain syndromes with autonomic testing demonstrated that patients with bladder and pelvic pain syndromes can experience orthostatic intolerance due to autonomic neuropathy [105]. Stenting of the iliac vein has also been shown to improve pelvic pain symptoms in POTS patients. In a previously mentioned large-scale retrospective study of patients with OI or POTS, iliac vein stenting for left iliac venous stenosis was associated with significant improvements in quality-of-life measures, as measured by multiple surveys (IPPS, PCS, Rome IBS, PUF, OHQ, and PGIC), at 3-month and 1-year follow-up [29]. In that study, OI patients exhibited symptoms of lower back pain, urinary symptoms, dysmenorrhea, and pelvic pain more commonly than lower extremity symptoms. Together, these findings support an association between pelvic pain and orthostatic symptoms in patients with POTS and underlying iliofemoral venous obstruction.

## 9. Chronic Lower Extremity Venous Disease

CVD is the downstream consequence of a spectrum of venous disorders that culminate in sustained venous hypertension. Patients commonly present with lower extremity edema, skin changes, venous ulcers, varicose veins, and sensory symptoms such as pain, itching, cramping, and heaviness [106,107]. CVD affects a substantial proportion of the global population, with prevalence estimates of 73% among women and 56% among men [108].

The pathophysiology of venous hypertension is multifactorial and includes elevated central venous pressure from volume overload, prior deep venous thrombosis, extrinsic venous compression, and valvular incompetence. Because the deep venous system accounts for approximately 90% of lower-extremity venous return, pathology at this level has disproportionate hemodynamic consequences [106]. Chronic venous hypertension leads to endothelial dysfunction, impaired venous capacitance regulation, and an increased risk of venous thromboembolism. These alterations may also limit the effectiveness of preload augmentation in upright posture, a key compensatory mechanism in orthostatic stress.

CVD may be suspected on bedside physical examination, but definitive diagnosis requires lower extremity duplex ultrasonography in the upright position to assess venous reflux, obstruction, abnormal anatomy, and flow dynamics. Hemodynamically significant reflux is defined as >0.5 s in the superficial venous system and >1 s in the deep venous system [109]. Management focuses on reducing venous hypertension and optimizing venous return, with first-line therapies including compression garments, leg elevation, and structured exercise programs emphasizing calf and foot flexion to enhance the skeletal muscle pump [106,110]. Diuretics are not indicated in CVD unless true volume overload is present, as inappropriate volume depletion may exacerbate OI in susceptible patients. In patients with refractory symptoms and concordant duplex findings, procedural interventions such as sclerotherapy, venous stripping, glue/adhesive, or endovascular therapies, including laser, radiofrequency, or chemical ablation, may be considered [106,110].

Venous insufficiency alone may contribute to autonomic dysfunction, while concomitant venous compression syndromes may contribute to OI by impairing venous capacitance and preload reserve based on limited mechanistic evidence. Emerging evidence links venous outflow obstruction to POTS, with improvement in orthostatic symptoms following compression garment therapy [111]. In a retrospective study of 191 female patients with POTS and venous insufficiency, significant left common iliac vein stenosis (>50%) was found in 69%. In contrast, only 40% of age-matched controls showed comparable compression, suggesting a higher prevalence of iliac venous obstruction and downstream venous insufficiency among female patients with POTS [112]. Some patients also show symptomatic improvement after treatment of pelvic or iliac venous disease. A case series of patients with POTS and chronic venous disease supported lower extremity radiofrequency ablation as a therapeutic strategy in POTS patients with refractory symptoms [113]. These findings support a potential contributory role of venous outflow limitation on OI in susceptible individuals, but higher-quality evidence is needed to clarify this association.

## 10. Overlap Between Vascular Compression Syndromes

Patients often present with more than one vascular compression syndrome, highlighting the complex relationships within the abdominal/pelvic vasculature [9,61]. In a retrospective study of 169 patients with abdominal compression syndromes, 72.8% had two or more syndromes, and 11.8% had up to four [9]. NCS and SMAS often co-occur because of their shared anatomy, with the LRV and duodenum coursing between the SMA and the aorta [114,115,116,117]. In addition, the literature reports the co-occurrence of MALS and NCS, and it has been hypothesized that compression of the celiac artery in MALS can lead to SMA dilation via collateral blood flow, thereby precipitating NCS [118,119]. Other studies have suggested that loss of mesenteric adiposity due to weight loss may also precipitate NCS and SMAS [56,119,120,121]. Overall, the coexistence of two or more compression syndromes is variable, and numerous case reports and series [21,56,118,120,121,122,123,124,125,126] describing overlapping syndromes warrant larger-scale studies to investigate whether there is shared pathophysiology.

## 11. Vascular Compression Syndromes and HSD

Numerous case studies and retrospective studies have linked vascular compression syndromes with HSD, although the direction and magnitude of the association have not been elucidated. Upper extremity compression syndromes have been associated with HSD in some studies, which is thought to result from alterations in connective tissue structure and function. Cerebral venous outflow disorders and HSD have been increasingly described, with IJVS being one of the most common sites of cerebral venous outflow impairment. A retrospective study of 86 patients suspected to have both cerebral venous outflow disorders and HSD found that 52% had IJVS [127]. Extrinsic IJV compression has also been shown to occur due to cervical instability, a consequence of connective tissue dysfunction seen in mild forms of HSD (52–66% with mild CI vs. 5% with severe CI) [31,128]. Patients with TOS may also have HSD. In the previously discussed study of patients presenting for evaluation of TOS or brachial plexus dysfunction, 58% had joint hypermobility defined by a Beighton score ≥ 4, and 42% had hEDS [2]. Additionally, a study evaluating the incidence of vTOS in patients with POTS identified that 70% patients also had EDS [129].

Abdominal and pelvic compression syndromes are also associated with HSD. A retrospective study of 169 patients with one or more abdominal vascular compression syndrome identified that 72.2% of patients also had a hypermobility-related disorder [9]. In contrast, another retrospective study of nearly 8000 patients with known HSD, mostly hEDS, identified compression syndromes in only 45 patients based on existing radiographic reports, suggesting minimal association [61]. The mechanism underlying the association between hEDS and abdominal and pelvic compression syndromes is unknown but has been hypothesized to involve vascular laxity, lordosis or scoliosis, celiac plexus inflammation, neurological causes, and gene variants [61].

Notably, hEDS is well-recognized for its association with autonomic nervous system dysfunction [10]. This association is thought to arise from underlying fascial and connective tissue abnormalities that compromise vascular, neurological, and structural integrity and impair normal remodeling processes [52]. Disease in these patients is also thought to be related to mast cell–mediated processes, histamine-driven processes, hyperadrenergic states, and the presence of autoimmune neural antibodies, but other pathophysiologic processes remain to be identified [10].

## 12. Discussion

This narrative review highlights a possible conceptual and clinical link between vascular compression syndromes, HSD, and dysautonomia. Each vascular compression syndrome has been linked with dysautonomia, whether theoretically or through small case series or larger cohort studies. The strongest associations are in MTS/NIV, as the literature demonstrates that interventional treatment improves OI symptoms [29]. Other associations, such as in NCS, are based on smaller cohort studies [3,7,8]. Some of these links are purely theoretical and anecdotal, as in IJVS and SMAS [1,32,43,44,77,78,79], which may be attributed to an absence of both prospective and retrospective data investigating this potential association. In SMAS in particular, the absence of association may also be attributable to its distinct pathophysiology, in that a luminal organ rather than a blood vessel is being compressed. These associations lack directionality and may be prone to bias, as discussed in the limitations section. However, this evidence highlights an important phenomenon and a novel area for prospective studies and randomized controlled trials, with the potential to significantly improve morbidity for this patient population.

The physiologic link between vascular compression syndromes and dysautonomia is plausible through pathways that influence preload, venous capacitance, autonomic reflexes, and sympathetic nerve irritation, although a conceptual framework integrating all these mechanisms remains theoretical. Moreover, other heterogeneous conditions, like HSD, mast cell activation disorder, and POTS, may cause similar symptoms together and separately, complicating our overall understanding of the disease process. The most verified mechanism is hemodynamic, in which venous outflow obstruction in various parts of the body can reduce effective venous return and limit preload reserve during orthostatic stress, leading to increased sympathetic activation to maintain cardiac output [5,35]. The evidence for this mechanism comes from improved orthostatic symptoms in patients with POTS following compression garment therapy [29,111], symptomatic improvement following interventions that augment venous return [113], and experimental evidence demonstrating increased autonomic signaling with occlusion of veins [102,103]. Other mechanisms remain largely theoretical and indirectly supported. In MALS, contemporary literature has determined that symptoms are largely neurogenic rather than ischemic, raising the possibility that celiac plexus irritation could amplify autonomic symptom burden [55,59]. While patient improvement with nerve blocks corroborates this mechanism, it is unclear whether the benefit is largely due to nociceptive analgesia or to improvement in true dysautonomia. Renovascular hypertension, altered renal hemodynamics, and impaired autonomic function have been described in pediatric patients with NCS and dysautonomia [3,7,8], but experimental evidence is lacking. Likewise, the proposed impaired venous outflow and increased ICP in patients with IJVS, accompanied by irritation of autonomic ganglia [1,32,43,44], is supported primarily by case-based literature rather than controlled physiological studies.

HSD are associated with dysautonomia and vascular compression syndromes in the literature, serving as both a condition with biological susceptibility to disease but also a condition subject to confounding in the methodology of some studies. HSD have a biological basis for compression and entrapment syndromes due to connective tissue laxity and altered fascial integrity. Alone, HSD are independently associated with autonomic dysfunction through neuropathic processes, mast cell disease, hyperadrenergic states, and autoimmune neural antibodies [10,52]. Orthostatic symptoms may exist alone in the absence of overt compressive lesions. This distinction is evident in the available literature. For example, a smaller cohort of patients with known vascular compression syndromes had a relatively high incidence of concomitant HSD, whereas another, larger cohort identified a small percentage of HSD patients with vascular compression syndromes through retrospective radiographic review [9,61]. This variable population prevalence is likely due to differences in the ascertainment strategies between the studies.

On a practical level, these findings suggest value in considering vascular compression syndromes in patients with dysautonomia or HSD. At minimum, we recommend evaluating patients with dysautonomia or HSD and symptoms of specific vascular compression syndromes (such as headache and sensory changes in IJVS, flank pain and hematuria in NCS, and lower extremity edema in CVD). While the causal association between vascular compression syndromes and dysautonomia symptoms is theoretical, in the future, there may be value in screening all patients with dysautonomia for these syndromes as more high-quality evidence emerges. These syndromes are not commonly recognized on routine imaging reads; therefore, they require particular attention on radiographic review. Proper ultrasound diagnosis is also sonographer-dependent, highlighting a need for training to recognize these underdiagnosed conditions. As there are no randomized controlled trials for the treatment of these syndromes, and studies are mostly limited to small single-center cohorts, treatment should be tailored in partnership with the individual patient. Invasive treatments carry procedural risks and should be considered after careful discussion with an interdisciplinary team, including interventional radiology and vascular surgery. Vascular intervention for dysautonomia alone without a clear anatomic or hemodynamic obstruction should remain investigational and individualized.

## 13. Limitations

The majority of evidence on the association between vascular compression syndromes, HSD, and dysautonomia is derived from small, single-center retrospective studies and case series or case reports. For example, evidence for an overlap between OI/POTS and NCS is limited to case series and case reports in pediatric patients, while adult data are largely absent. Unfortunately, there are no prospective studies addressing these associations, nor are there any randomized controlled trials investigating treatment modalities for vascular compression syndromes and their effects on dysautonomia symptoms. In addition, many of these studies are conducted in subspecialty clinics, which predisposes to selection and referral bias. Publication bias may also distort the associations found. Finally, many treatment studies have short-term follow-up, and there is limited data regarding the long-term effects of treatments for vascular compression syndromes. High-quality research is further limited by a lack of consensus on specific diagnostic thresholds for the different vascular compression syndromes. That said, there is a body of intervention-related literature describing improvement in OI symptoms in larger cohorts of patients with MTS/NIVL, providing higher-quality data on MTS/NIVL, specifically. The growing body of evidence, albeit with significant limitations, for these associations underscores the need for prospective studies and randomized controlled trials in this area.

## 14. Conclusions

Vascular compression syndromes represent a heterogeneous group of underrecognized and underdiagnosed conditions that may contribute to symptom burden in patients with dysautonomia. The literature reviewed in this study highlights a recurring overlap between vascular compression syndromes and manifestations of autonomic dysfunction such as OI and POTS. Despite this overlap, a key theme throughout the literature is the limited quality and heterogeneity of available data, with diagnostic criteria for dysautonomia and vascular compression syndromes varying widely across studies and frequently lacking standardized thresholds. Nevertheless, insights from the available literature include possible shared biomechanical factors in multiple compressive processes, hemodynamic mechanisms of compression, and neurogenic mechanisms involving autonomic ganglia. Taken together, vascular compression syndromes represent an important but unexplored contributor to autonomic symptomatology in selected patient populations. Our intention is to highlight these syndromes to clinicians, ensuring they are considered when providing care to this underserved patient population.

## 15. Future Directions

Prospective research is necessary to assess the prevalence of vascular compression syndromes in both dysautonomia and HSD populations. Furthermore, there exists a paucity of research examining the effects of treating vascular compression syndromes specifically on dysautonomia. Currently, no randomized controlled trials have been conducted to evaluate the impact of stenting and other interventions on the symptoms of dysautonomia in patients with compression syndromes. Such work will be critical for clarifying the pathophysiologic relationships between vascular anatomy and autonomic function and for determining the clinical relevance of vascular compression syndromes in patients with dysautonomia and HSD.

## Figures and Tables

**Table 1:** Key Features and Diagnostic Imaging Findings for Vascular Compression Syndromes.

Compression Syndrome	Key Features	Diagnostic Imaging
Internal Jugular Venous Stenosis	Symptomatic external compression of the IJV with increased intracranial pressure and venous hypertension	Radiograph: Use of lateral cranial and cervical x-ray can identify elongated styloid processes or degenerative changes of the spine (suggestive, non-diagnostic) CTV and MRV: No consensus on clinically significant jugular venous compression, approximately >50% cross-sectional area reduction is standard across studies on interventions [12,13] (context-dependent) Can identify anatomical relationships to the vessel tract and can identify focal areas of stenosis and vessel collateralization as well as parenchymal changes related to chronic venous hypertension [14,15] (suggestive, non-diagnostic)
Thoracic Outlet Syndrome	Symptomatic compression of the thoracic outlet and neurovascular structures due to musculoskeletal abnormalities and/or overuse injury, resulting in pain and decreased function of the affected side	Radiograph: Chest x-ray to evaluate for underlying structural abnormalities (i.e., cervical rib) [16] (suggestive, non-diagnostic) Doppler Ultrasonography: Limited utility, but can identify flow abnormalities with position changes (suggestive, non-diagnostic) MRI and MRA/MRV: Useful in the evaluation of vTOS and nTOS given visual resolution, obtained in abducted and adducted positions to evaluate for reduced blood flow and brachial plexus abnormalities [17,18] (widely accepted) MRI may show brachial plexus edema and loss of fat about the brachial plexus in fixed abduction [17] (suggestive, non-diagnostic) MRA/MRV will show stenosis, enlarged collateral circulation, and possible thrombosis at the site of narrowing [17] (suggestive, context-dependent)
Median Arcuate Ligament Syndrome	Symptomatic compression of the celiac trunk and plexus by the MAL, resulting in nausea and vomiting, anorexia, and abdominal pain	Doppler Ultrasonography: Celiac trunk peak systolic velocity during expiration > 200 cm/s or end diastolic velocity > 350 cm/s [19,20] (suggestive, context-dependent) Celiac trunk deflection angle > 50° (end-expiratory) [19,20] (suggestive, context-dependent) Celiac trunk-to-aorta ratio (peak systolic velocity) > 3:1 [20] (suggestive, context-dependent) CTA: Demonstrates vessel collateralization, proximal celiac trunk narrowing, and post-stenotic dilation with prominent downstream arteries (suggestive, non-diagnostic) Vessel distortion is not necessary for the diagnosis of neurogenic MALS. Soft tissue from the MAL on the anterior/ superior surface of the celiac artery on sagittal imaging may suggest benefit from celiac plexus block. (suggestive, context-dependent) MAL thickness > 4 mm is abnormal and suggestive of pathology [21,22] (widely accepted)
Superior Mesenteric Artery Syndrome	Symptomatic compression of the duodenum between the SMA and the aorta, resulting in duodenal obstruction, vomiting, dyspepsia, epigastric pain, and early satiety.	Doppler Ultrasonography: Reduced aortic-mesenteric angle < 25° and distance < 8–10 mm [22] (widely accepted) CTA and MRA: Aortic-mesenteric angle < 22° and aorto-mesenteric distance < 8 mm [21,23] (widely accepted) Duodenal compression and proximal dilation with gastric distension and a proximal SMA cutoff point [21,23] (suggestive, context-dependent)
Nutcracker Syndrome	Symptomatic compression of the LRV between the SMA and the aorta, causing flank pain, hematuria, varicocele or ovarian vein varices, and pelvic pain	Radiographic findings are suggestive of a diagnosis but not definitive, and they are similar with most modalities (US, CT, MRI, angiography): Reduced aortic-SMA angle < 38° (38–65° normal range) (suggestive, context-dependent) LRV stenosis with proximal dilation and >32° dilation angle between the SMA and aorta (“beak sign”) [21,22] (widely accepted) Evidence of vessel collateralization in the paralumbar, epidural, gonadal, phrenic, and intercostal veins (suggestive, non-diagnostic) LRV peak flow ratio 4–5 (ratio of flow on Doppler ultrasound between compressed and non-compressed portion of LRV) [24] (suggestive, non-diagnostic) LRV to IVC pressure gradient > 3 mmHg [23] (suggestive, non-diagnostic) Compression ratio > 2.25 (Anteroposterior diameter of pre-compressed LRV to compressed LRV) is both sensitive and specific for NCS [25] (limited data)
May-Thurner Syndrome/Non-Thrombotic Iliac Vein Lesion	Symptomatic extrinsic compression of the LCIV by an overlying RCIA and lumbar vertebral body, resulting in pelvic pain, unilateral leg edema, erythema, warmth, and pain	Ultrasonography: Doppler ultrasound utilized as the initial screening rest for stenosis and thrombosis detection, although limited evaluation of pelvic iliac veins (suggestive, non-diagnostic) IVUS and Venography IVUS is the gold-standard for evaluation given 360° internal view with high resolution images of the vessel wall at the compression site and in the normal venous segments allowing for accurate sizing and comparison to normal segments (widely accepted) Limited data suggest >50% luminal stenosis as a threshold for significant disease that would benefit from intravascular stenting in thrombotic disease [26], while a subset analysis suggests benefit in non-thrombotic patients with >61% stenosis by diameter [27] (limited data) Other data suggest 50% compression by cross-sectional area as a cutoff in pelvic pain patients [28] (limited data) CTV and MRV: Comprehensive evaluation of the pelvic and abdominal vasculature and masses that may cause extrinsic compression (suggestive, non-diagnostic) Visualization of collateral vessel formation and pelvic venous congestion that suggest iliofemoral disease [24] (suggestive, non-diagnostic) Ability to assess for thrombotic and nonthrombotic disease [23] (suggestive, context-dependent) CTV has been shown in limited data to be less sensitive in iliac vein compression than CT when compared to venography and IVUS [29] (limited data)

Abbreviations: *IJV*, Internal Jugular Vein; *CTV*, Computed Tomography Venography; *MRV*, Magnetic Resonance Venography; *CTA*, Computed Tomography Angiography; *MRA*, Magnetic Resonance Angiography; *IVUS*, Intravascular Ultrasound; *vTOS*, Vascular Thoracic Outlet Syndrome; *nTOS*, Neurogenic Thoracic Outlet Syndrome; *MAL*, Median Arcuate Ligament; *SMA*, Superior Mesenteric Artery; *LRV*, Left Renal Vein; *IVC*, Inferior Vena Cava; NCS, Nutcracker Syndrome; *LCIV*, Left Common Iliac Vein; *RCIA*, Right Common Iliac Artery.

## Data Availability

No new data were created or analyzed in this study. Data sharing is not applicable to this article.

## Abbreviations

The following abbreviations are used in this manuscript:

IJVS	Internal jugular venous stenosis
ICP	Intracranial pressure
EBV	Epstein-Barr virus
TOS	Thoracic outlet syndrome
nTOS	Neurogenic thoracic outlet syndrome
vTOS	Venous thoracic outlet syndrome
aTOS	Arterial thoracic outlet syndrome
MALS	Median arcuate ligament syndrome
MAL	Median arcuate ligament
NCS	Nutcracker syndrome
NCP	Nutcracker phenomenon
SMAS	Superior mesenteric artery syndrome
SMA	Superior mesenteric artery
LRV	Left renal vein
POTS	Postural orthostatic tachycardia syndrome
OI	Orthostatic intolerance
HSD	Hypermobility spectrum disorders
hEDS	Hypermobile Ehlers-Danlos syndrome
EAST	Elevated arm stress test
MTS	May-Thurner syndrome
NIVL	Non-thrombotic iliac vein lesion
DVT	Deep venous thrombosis
IVUS	Intravascular ultrasound
